# Cross-cultural adaptation of the *Escala De Fatigabilidad Vocal en Docentes (EFV-D)* to brazilian portuguese

**DOI:** 10.1590/2317-1782/e20250426en

**Published:** 2026-08-17

**Authors:** Maria Eduarda Oliveira de Figueiredo, Priscila Oliveira, Thays Vaiano, Thiago Henrique de Pontes Ferreira, Larissa Nadjara Alves Almeida

**Affiliations:** 1 Departamento de Fonoaudiologia, Universidade Federal da Paraíba – UFPB - João Pessoa (PB), Brasil.; 2 Centro de Estudos da Voz – CEV - São Paulo (SP), Brasil.; 3 Faculdade IDE – Sergipe (SE), Brasil.

**Keywords:** Voice, Voice Disorders, Fatigue, Faculty, Diagnostic Self Evaluation, Validation Study

## Abstract

**Purpose:**

To perform the cross-cultural adaptation of the Vocal Fatigability Scale for Teachers (VFS-T) to Brazilian Portuguese (BP).

**Method:**

The procedures followed the recommendations of Beaton et al. and the guidelines of the International Test Commission (ITC). Originally developed and validated in Spanish, the EFV-D assesses vocal fatigability in teachers. It consists of 17 items divided into two factors: 11 related to sensations during voice use and six related to recovery strategies and limitations due to excessive vocal use. The process comprised five stages: (1) translation into BP by two speech-language pathologists and one sworn translator, all Brazilian and fluent in Spanish; (2) synthesis of the translations; (3) pre-testing with 23 elementary, secondary, and higher education teachers, who evaluated the relevance of the items; (4) back-translation performed by one speech-language pathologist and one non-specialist translator; and (5) consensus of the versions and development of the final version by an expert committee.

**Results:**

During translation, adjustments were required in the title of factor 2 and all items for better interpretation, with 15 items reformulated and two content-adjusted. In the back-translation, there were structural discrepancies in 14 items and content discrepancies in 3, which were resolved by the expert committee. Overall, teachers assigned satisfactory levels of importance, clarity, and interpretation to most items. Those with a relevance index below 50% were modified according to equivalence criteria.

**Conclusion:**

The cross-cultural adaptation of the EFV-D into PB resulted in the final version entitled the Vocal Fatigability Scale for Teachers (EFV-P). Future studies should be conducted to verify its validity and reliability.

## INTRODUCTION

​Teachers represent the group of voice professionals most affected by vocal disorders, as the voice constitutes their primary work tool, making them particularly susceptible to the development of dysphonia^(1)^. Environmental and organizational aspects of teaching, including excessive noise, dust exposure, long working hours, and task overload, contribute to impaired vocal health. Excessive workload increases phonatory demands and overloads the vocal apparatus, favoring the emergence of vocal symptoms and disorders^(2)^. Among the symptoms most frequently reported by teachers are muscular tension, vocal effort, reduced vocal projection, hoarseness, and fatigue, the latter being the most recurrent physical complaint^(3)^.

Vocal fatigue is characterized by self-perceived symptoms manifested through increased phonatory effort in response to elevated vocal demand. These symptoms generally improve following adequate vocal rest. Currently, the definition of vocal fatigue encompasses two major manifestations: performance fatigue and perceived fatigue^(4,5)^.

Performance fatigue refers to a decline in vocal quality over a specific period and is associated with three main factors: the viscoelastic properties of the vocal folds, the contractile capacity of the muscles involved in phonation, and the ability of the nervous system to provide adequate activation signals through the afferent and efferent pathways responsible for regulating vocal tasks^(4,5)^.

​In contrast, perceived fatigue may occur during or after vocal activity. This perception is influenced by modulatory factors such as mood, motivation, pain, and individual feedback mechanisms, functioning as an indicator that vocal rest is necessary^(4,5)^.

Occasional vocal fatigue may result in isolated and spontaneously reversible damage to the phonatory system when adequate rest is provided. However, recurrent vocal fatigue, referred to as “vocal fatigability” by Contreras-Regatero and Vila-Rovira (2024), corresponds to a generalized sensation of fatigue sustained over prolonged periods and resulting from the interaction between performance fatigue and perceived fatigue. In this condition, which extends beyond isolated and episodic manifestations, continuous tissue damage occurs and the healing process remains in a persistent state of repair, potentially preventing complete recovery^(5,6)^. As a broader construct, vocal fatigability enables a more comprehensive understanding of the impact of fatigue on participation in daily activities and on quality of life^(5)^.

The assessment and measurement of vocal fatigability require an understanding of the patient’s self-perception regarding this phenomenon. Such information can be obtained through vocal self-assessment, one of the main components of multidimensional voice assessment. Vocal self-assessment is conducted through instruments composed of specific items that provide essential information for vocal diagnosis, including aspects that cannot be identified through other evaluative methods. The absence of the patient’s perspective renders voice assessment incomplete^(7)^.

Self-assessment instruments must be appropriate for the construct and population under investigation to ensure valid interpretations and accurate measurement of the intended construct. In addition, these instruments should demonstrate stability, precision, and fairness, allowing for unbiased assessment across individuals^(8)^.

​Several instruments have been developed for teachers, such as the Vocal Production Condition - Teacher (CPV-T), which characterizes teachers’ vocal profiles and occupational conditions, and the Voice Disorder Screening Index (ITDV), designed to identify individuals at risk for vocal disorders^(9,10)^. Additionally, the Voice Disorder Screening Index (VDSI) contributes to the identification of vocal fatigue; however, it does not specifically assess vocal sensations in relation to the demanding vocal activities that trigger them^(11)^. Therefore, although these instruments are directed toward the teaching population, they do not specifically assess vocal fatigability. In contrast, the *Escala* de *Fatigabilidad Vocal* en*Docentes* (EFV-D) investigates the relationship between vocal fatigability and teachers’quality of life, specifically associating vocal sensations with the vocal demands that elicited them^(5,6)^.

Considering that vocal fatigability is a frequent symptom among teachers, the use of a specific and sensitive instrument capable of addressing its characteristics, such as the EFV-D, becomes essential. Developed and validated in Spanish in the city of Barcelona, the EFV-Daims to detect vocal fatigability in teachers. The instrument comprises 17 items divided into two factors: 11 items related to sensations during voice use and six items associated with recovery strategies and limitations resulting from excessive vocal use^(6)^.

The investigation of vocal fatigability in Brazilian teachers using the EFV-D will be made possible through its cross-cultural adaptation and validation for Brazilian Portuguese and culture. This process will enable the collection of relevant data for speech-language pathology assessment and intervention. Although the original validation study of the scale included a free translation into Brazilian Portuguese, it did not follow the content validation procedures recommended by international guidelines. Therefore, the present study aimed to perform the cross-cultural adaptation of the EFV-D into Brazilian Portuguese (BP).

## METHODS

​This was a cross-sectional methodological study approved by the Research Ethics Committee under protocol number 7,406,255. The study followed the ethical principles established by CNS Resolution 466/12, and all participants signed an Informed Consent Form (ICF).

The present study focused on the cross-cultural adaptation of the *Escala de Fatigabilidad Vocal en Docentes (EFV-D*) for the Brazilian teaching population. The aim was to linguistically and culturally adapt the scale, ensuring its conceptual equivalence and appropriateness to the Brazilian sociocultural context, thereby enabling its application as a sensitive and specific instrument for the detection of vocal fatigability in Brazilian teachers. The procedures adopted for the cross-cultural adaptation of the EFV-D into Brazilian Portuguese (BP) followed the recommendations proposed by Beaton et al.^(12)^ and the guidelines of the *International Test Commission* (ITC)^(13)^.

The EFV-D was originally developed and validated in Spanish in the city of Barcelona. The scale was designed to assess levels of vocal fatigability in teachers and to analyze variations according to personal and occupational characteristics. The instrument comprises 17 items distributed across two factors: Factor 1 - Fatigue in vocal performance, consisting of 11 items (items 1 to 11), which assess sensations and perceptions associated with fatigue during voice use; and Factor 2 - Reparative behaviors, composed of six items (items 12 to 17), which investigate strategies used for vocal recovery. Each item is rated using a five-point Likert scale: 0 = Never; 1 = Almost never; 2 = Sometimes; 3 = Almost always; and 4 = Always. The total score is obtained through the simple summation of all item scores. Teachers’ levels of vocal fatigability are classified into three categories according to the proposed cutoff points: no fatigability (score <15), moderate fatigability (score between 15 and 27), and high fatigability (score >28)^(6)^.

​The cross-cultural adaptation process of the EFV-D into BP consisted of five stages: (1) translation into Brazilian Portuguese; (2) synthesis of the translated versions; (3) pre-testing; (4) back-translation; and (5) consensus of the back-translated versions and development of the final version. All stages were conducted virtually. For the adaptation process, the title, factors, and all scale items were considered.

In the translation stage (Stage 1), the instrument was independently translated by three translators, two speech-language pathologists and one sworn translator, all native speakers of Brazilian Portuguese and fluent in Spanish. Translators received the original Spanish version and were instructed to prioritize cultural equivalence during translation. Subsequently, the expert committee, composed of the study authors, developed by consensus a synthesized version of the translations (Stage 2), resulting in a single version of the title, factors, and items. This synthesized version was then submitted to the pre-testing stage (Stage 3) to conduct a preliminary evaluation of teachers’ understanding and applicability of the scale among professionals actively engaged in teaching at the time of the study.

Teachers participating in the pre-test were recruited virtually through an invitation containing a link to the Google Forms platform. In the online form, participants had access to the Informed Consent Form, the transculturally adapted version of the scale, and the response instructions. Participants were instructed to evaluate each item according to the following response options: 1 = Item not relevant; 2 = Item requiring revision to assess relevance; 3 = Item relevant but requiring minor modifications; and 4 = Item absolutely relevant. Participants were also encouraged to suggest modifications for items considered culturally inappropriate or difficult to understand. After data collection, responses were tabulated in a spreadsheet and analyzed using relative frequency measures. Items that obtained a relevance index equal to or lower than 50% were revised. Participants’ suggestions were analyzed and accepted when considered appropriate.

​The sample consisted of 23 teachers: six elementary school teachers (26.1%), nine secondary school teachers (39.1%), and eight higher education teachers (34.8%). Participants’ ages ranged from 23 to 65 years, with a mean age of 39 years and 7 months (SD = 13.48). Eligibility criteria included being a teacher aged 18 years or older, being a native speaker of Brazilian Portuguese, and currently working in elementary, secondary, or higher education.

The version established after the pre-test was subsequently submitted to back-translation into the source language (Stage 4) by two translators fluent in Spanish and native speakers of Brazilian Portuguese, one speech-language pathologist and one Spanish language teacher, with the purpose of identifying possible changes in the original content. The expert committee then prepared, by consensus, a synthesis of the back-translated versions, which was compared and analyzed in relation to the original EFV-D version, considering semantic, idiomatic, conceptual, linguistic, experiential, and contextual equivalences (Stage 5). Based on this analysis, the final version of the scale transculturally adapted into Brazilian Portuguese was established (Appendix 1).

Data regarding teachers’ assessments of the relevance of the items in the scale, then entitledEscala de Fatigabilidade Vocal em Professores (EFV-P), were analyzed quantitatively using descriptive statistics, including absolute and relative frequency measures, as well as inferential analysis using the proportion comparison test. Responses were categorized as “Item not relevant,” “Item requiring revision to assess relevance,” “Item relevant but requiring minor modifications,” and “Item absolutely relevant,” according to the response options provided in the scale. Statistical analyses were conducted using the *Statistical Package* for *the Social Sciences* (SPSS), version 25.0.0, adopting a significance level of 5% (p < 0.05).

## RESULTS

​During the translation stage, there was agreement among the translators regarding the name of the instrument, Escala de Fatigabilidade Vocal em Docentes (EFV-D), and the title of Factor 1, Fadiga no rendimento vocal. However, disagreement emerged regarding the title of Factor 2, requiring adjustment by the expert committee, which established the final version as Condutas reparadoras.

Regarding the translation of the items, the translators proposed different wording for 15 statements, specifically items 1, 2, 3, 4, 5, 6, 7, 9, 10, 12, 13, 14, 15, 16, and 17. In items 8 and 11, disagreements involved conceptual content. For item 8, the proposed versions were: “Depois de mais de 2 horas falando em forte intensidade, sinto minha voz irritada”; “Depois de mais de 2 horas falando em alta intensidade, sinto a garganta irritada”; and “Depois de mais de 2 horas falando em alta intensidade, sinto a voz irritada”. For item 11, the suggested versions were: “Sinto desconforto ou dor no pescoço depois de um dia inteiro usando a voz”; “No final do dia, sinto incômodo ou dor na garganta usando minha voz”; and “Sinto desconforto ou dor no pescoço ao final de um dia usando a voz”. Overall, inconsistencies were observed regarding the use of the terms “forte” and “alta” to refer to vocal intensity.

During the synthesis of the translated versions, the expert committee maintained items 1, 2, 3, 7, 11, 13, 15, 16, and 17 according to at least one of the translated versions. Items 4, 5, 6, 8, 9, 10, 12, and 14 were modified to improve linguistic appropriateness and comprehensibility for the target culture and population (Chart 1). Adjustments were made to items that presented a relevance index equal to or lower than 50%, in accordance with equivalence criteria and considered the suggestions provided by the teachers. The term “forte” was ultimately maintained when referring to vocal intensity.

**Chart 1:** Results of the cross-cultural adaptation process of the EFV-D into Brazilian Portuguese.

**Versão original**	**Tradução**	**Síntese**	**Retrotradução**	**Versão final**
**Título**				
Escala de Fatigabilidad Vocal en Docentes (EFV-D)	T1: Escala de Fatigabilidade Vocal em Docentes (EFV-D)	Escala de Fatigabilidade Vocal em Docentes (EFV-D)	R1: Escala de Fatigabilidad Vocal en Docentes (EFV-D)	Escala de Fatigabilidade Vocal em Docentes (EFV-D)
	T2: Escala de Fatigabilidade Vocal em Docentes (EFV-D)			
			R2: Escala de Fatigabilidad Vocal en Docentes (EFV-D)	
	T3: Escala de Fatigabilidade Vocal em Docentes (EFV-D)			
**Fator 1**				
Fatiga en el rendimiento vocal	T1: Fadiga no rendimento vocal	Fadiga no rendimento vocal	R1: Fatiga en el rendimiento vocal	Fadiga no rendimento vocal
	T2:Fadiga no rendimento vocal			
			R2: Fatiga en el rendimiento vocal	
	T3: Fadiga no rendimento vocal			
**Fator 2**				
Conductas reparadoras	T1: Condutas reparadoras	Condutas reparadoras	R1: Conductas reparadoras	Condutas reparadoras
	T2: Hábitos de recuperação vocal			
			R2: Conductas reparadoras	
	T3: Comportamentos reparadores			
**Itens**				
1 - Después de más de 2 horas hablando a una intensidad alta no puedo seguir con tareas vocales exigentes.	T1: Depois de mais de 2 horas falando em forte intensidade, não consigo continuar em tarefas de grande exigência vocal.	Depois de mais de 2 horas falando em forte intensidade, não consigo continuar em tarefas de grande exigência vocal.	R1: Después de más de 2 horas hablando con alta intensidad, no puedo continuar con tareas que exigen mucho esfuerzo vocal.	Depois de mais de 2 horas falando em forte intensidade, não consigo continuar em tarefas de grande exigência vocal.
	T2: Depois de mais de 2 horas falando em alta intensidade, não posso continuar com tarefas vocais intensas.			
			R2: Después de más de 2 horas hablando con fuerte intensidad, no puedo seguir las tareas de gran exigencia vocal.	
	T3: Depois de mais de 2 horas falando em alta intensidade, não consigo continuar com tarefas vocais exigentes.			
2 - Después de más de 2 horas hablando a una intensidad alta siento la voz cansada.	T1: Depois de mais de 2 horas falando em forte intensidade, sinto a voz cansada.	Depois de mais de 2 horas falando em forte intensidade, sinto a voz cansada.	R1: Después de más de 2 horas hablando con alta intensidad, siento la voz cansada.	Depois de mais de 2 horas falando em forte intensidade, sinto a voz cansada.
	T2: Depois de mais de 2 horas falando em alta intensidade, sinto a voz cansada.			
			R2: Después de más de 2 horas hablando con fuerte intensidad, siento la voz cansada.	
	T3: Depois de mais de 2 horas falando em alta intensidade, sinto a voz cansada.			
3 - Después de más de 2 horas hablando a una intensidad alta experimento una creciente sensación de esfuerzo en la voz.	T1: Depois de mais de 2 horas falando em forte intensidade, percebo uma crescente sensação de esforço vocal.	Depois de mais de 2 horas falando em forte intensidade, percebo uma crescente sensação de esforço vocal.	R1: Después de más de 2 horas hablando con alta intensidad, noto una creciente sensación de esfuerzo vocal.	Depois de mais de 2 horas falando em forte intensidade, percebo uma crescente sensação de esforço vocal.
	T2: Depois de mais de 2 horas falando em alta intensidade, sinto uma crescente sensação de esforço na minha voz.			
			R2: Después de más de 2 horas hablando con fuerte intensidad, me doy cuenta de una creciente sensación de esfuerzo vocal.	
	T3: Depois de mais de 2 horas falando em alta intensidade, experimento uma sensação crescente de esforço na voz.			
4 - Después de más de 2 horas hablando a una intensidad alta mi voz se vuelve ronca.	T1: Depois de mais de 2 horas falando em forte intensidade, fico com a voz rouca.	Depois de mais de 2 horas falando em forte intensidade minha voz fica rouca.	R1: Después de más de 2 horas hablando con alta intensidad, mi voz se vuelve ronca.	Depois de mais de 2 horas falando em forte intensidade, minha voz fica rouca.
	T2: Depois de mais de 2 horas falando em alta intensidade minha voz fica rouca.			
			R2: Después de más de 2 horas hablando con fuerte intensidad, mi voz se vuelve ronca.	
	T3: Depois de mais de 2 horas falando em alta intensidade, minha voz fica rouca.			
5 - Después de más de 2 horas hablando a una intensidad alta me resulta un esfuerzo seguir hablando como lo estaba haciendo.	T1: Depois de mais de 2 horas falando em forte intensidade, tenho que fazer esforço para seguir falando como antes.	Depois de mais de 2 horas falando em forte intensidade, tenho que fazer esforço para continuar falando como estava fazendo.	R1: Después de más de 2 horas hablando con alta intensidad, tengo que hacer un esfuerzo para seguir hablando como lo estaba haciendo.	Depois de mais de 2 horas falando em forte intensidade, tenho que fazer esforço para continuar falando como estava fazendo.
	T2: Depois de mais de 2 horas falando em alta intensidade é um esforço para continuar falando como eu estava fazendo.			
			R2: Después de más de 2 horas hablando con fuerte intensidad, tengo que esforzarme para continuar hablando como lo estaba haciendo.	
	T3: Depois de mais de 2 horas falando em alta intensidade, é um esforço continuar falando como estava.			
6 - Después de más de 2 horas hablando a una intensidad alta me resulta difícil seguir proyectando la voz.	T1: Depois de mais de 2 horas falando em forte intensidade, sinto dificuldade para projetar a voz.	Depois de mais de 2 horas falando em forte intensidade, sinto dificuldade para continuar projetando a voz.	R1: Después de más de 2 horas hablando con alta intensidad, siento dificultad para seguir proyectando la voz.	Depois de mais de 2 horas falando em forte intensidade, sinto dificuldade para continuar projetando a voz.
	T2: Depois de mais de 2 horas falando em alta intensidade, torna-se difícil continuar projetando a voz.			
			R2: Después de más de 2 horas hablando con fuerte intensidad, siento dificultad para seguir proyectando la voz.	
	T3: Depois de mais de 2 horas falando em alta intensidade, é difícil continuar projetando a voz.			
7 - Después de más de 2 horas hablando a una intensidad alta siento mi voz débil.	T1: Depois de mais de 2 horas falando em forte intensidade, sinto minha voz fraca.	Depois de mais de 2 horas falando em forte intensidade, sinto minha voz fraca.	R1: Después de más de 2 horas hablando con alta intensidad, siento mi voz débil.	Depois de mais de 2 horas falando em forte intensidade, sinto minha voz fraca.
	T2: Depois de mais de 2 horas falando em alta intensidade, sinto minha voz fraca.			
			R2: Después de más de 2 horas hablando con fuerte intensidad, siento mi voz débil.	
	T3: Depois de mais de 2 horas falando em alta intensidade, sinto minha voz fraca.			
8 - Después de más de 2 horas hablando a una intensidad alta siento la voz irritada.	T1: Depois de mais de 2 horas falando em forte intensidade, sinto minha voz irritada.	Depois de mais de 2 horas falando em forte intensidade, sinto a garganta irritada.	R1: Después de más de 2 horas hablando con alta intensidad, siento dolor de garganta.	Depois de mais de 2 horas falando em forte intensidade, sinto a garganta irritada.
	T2: Depois de mais de 2 horas falando em alta intensidade, sinto a garganta irritada.			
			R2: Después de más de 2 horas hablando con fuerte intensidad, siento la garganta irritada.	
	T3: Depois de mais de 2 horas falando em alta intensidade, sinto a voz irritada.			
9 - Después de más de 2 horas hablando a una intensidad alta siento incomodidad en el cuello, la garganta o la lengua.	T1: Depois de mais de 2 horas falando em forte intensidade, sinto incomodo no pescoço, garganta ou língua.	Depois de mais de 2 horas falando em forte intensidade, sinto desconforto no pescoço, na garganta ou na língua.	R1: Después de más de 2 horas hablando con alta intensidad, siento malestar en el cuello, la garganta o la lengua.	Depois de mais de 2 horas falando em forte intensidade, sinto desconforto no pescoço, na garganta ou na língua.
	T2: Depois de mais de 2 horas falando em alta intensidade sinto desconforto no pescoço, na garganta ou na língua.			
			R2: Después de más de 2 horas hablando con fuerte intensidad, siento molestia en el cuello, en la garganta y en la lengua.	
	T3: Depois de mais de 2 horas falando em alta intensidade, sinto desconforto no pescoço, na garganta ou na língua.			
10 - Después de más de 2 horas hablando a una intensidad alta siento dificultad para hablar con voz suave.	T1: Depois de mais de 2 horas falando em forte intensidade, sinto dificuldade para falar baixo.	Depois de mais de 2 horas falando em forte intensidade, sinto dificuldade para falar baixo/suave.	R1: Después de más de 2 horas hablando con alta intensidad, me cuesta hablar en voz baja.	Depois de mais de 2 horas falando em forte intensidade, sinto dificuldade para falar baixo.
	T2: Depois de mais de 2 horas falando em alta intensidade, sinto dificuldade em falar com uma voz suave.			
			R2: Después de más de 2 horas hablando con fuerte intensidad, siento dificultad para hablar en voz baja.	
	T3: Depois de mais de 2 horas falando em alta intensidade, tenho dificuldade em falar com voz suave.			
11 - Siento malestar o dolor en el cuello al final de un día utilizando la voz.	T1: Sinto desconforto ou dor no pescoço depois de um dia inteiro usando a voz.	Depois de um dia inteiro usando a voz, sinto desconforto ou dor no pescoço.	R1: Después de un día entero usando la voz, siento malestar vocal o dolor en el cuello.	Depois de um dia inteiro usando a voz, sinto desconforto ou dor no pescoço.
	T2: No final do dia, sinto incômodo ou dor na garganta usando minha voz.			
			R2: Después de un día entero usando la voz, siento malestias o dolor en el cuello.	
	T3: Sinto desconforto ou dor no pescoço ao final de um dia usando a voz.			
12 - Generalmente tiendo a limitar el habla después de un periodo de tiempo usando la voz.	T1: Geralmente limito a quantidade de fala depois de um tempo utilizando a voz.	Depois de um tempo utilizando a voz, costumo limitar a quantidade de fala.	R1: Tras un período de uso continuo de la voz, reduzco mi habla para evitar fatiga.	Depois de um tempo utilizando a voz, costumo limitar a quantidade de fala.
	T2: Geralmente costumo limitar a fala depois de um período de tempo considerável usando a voz.			
			R2: Después de un tiempo usando la voz, tiendo a limitar el uso del habla.	
	T3: Geralmente, tenho a tendência de limitar a fala após um período utilizando a voz.			
13 - Evito situaciones sociales en las que sé que tengo que hablar más.	T1: Evito situações sociais em que sei que preciso falar mais.	Evito situações sociais nas quais eu sei que vou falar muito.	R1: Evito situaciones sociales en las que sé que tendré que hablar mucho.	Evito situações sociais em que sei que vou falar muito.
	T2: Evito situações sociais nas quais eu sei que vou falar muito.			
			R2: Evito situaciones sociales en las que sé que voy a hablar mucho.	
	T3: Evito situações sociais nas quais sei que precisarei falar mais.			
14 - Siento que no puedo hablar con mi familia después de un día de trabajo.	T1: Sinto que não consigo falar com minha família depois de um dia de trabalho.	Depois de um dia de trabalho, sinto que não consigo falar com minha família.	R1: Al terminar mi jornada laboral, no tengo energía para hablar con mi familia.	Sinto que não consigo falar com minha família depois de um dia de trabalho.
	T2: Sinto que não posso falar com minha família depois de um dia de trabalho.			
			R2: Siento que no puedo hablar con mi familia después de un día de trabajo.	
	T3: Sinto que não consigo falar com minha família após um dia de trabalho.			
15 - Necesito guardar silencio e hidratarme más de 1 h para dejar de sentir sobreesfuerzo al hablar.	T1: Preciso ficar em silêncio e me hidratar por mais de 1 hora para deixar de sentir esforço para falar.	Preciso ficar em silêncio e me hidratar por mais de 1 hora para deixar de sentir esforço para falar.	R1: Necesito quedarme en silencio e hidratarme por más de 1 hora para dejar de sentir esfuerzo al hablar.	Preciso ficar em silêncio e me hidratar por mais de 1 hora para deixar de sentir esforço para falar.
	T2: Preciso ficar em silêncio e me hidratar por mais de 1 hora para parar de sentir esforço excessivo ao falar.			
			R2: Necesito estar en silencio e hidratarme durante más de 1 hora para dejar de sentir el esfuerzo de hablar.	
	T3: Preciso ficar em silêncio e me hidratar por mais de 1 hora para deixar de sentir sobrecarga ao falar.			
16 - Necesito dormir toda una noche para dejar de sentir malestar al hablar.	T1: Preciso dormir uma noite inteira para deixar de sentir desconforto ao falar.	Preciso dormir uma noite inteira para parar de sentir desconforto ao falar.	R1: Después de sentir malestar al hablar, necesito dormir una noche entera para recuperar la plenitud de mi voz.	Preciso dormir uma noite inteira para deixar de sentir desconforto ao falar.
	T2: Preciso dormir a noite toda para parar de sentir desconforto ao falar.			
			R2: Necesito dormir una noche entera para dejar de sentir malestar al hablar.	
	T3: Preciso dormir uma noite inteira para parar de sentir desconforto ao falar.			
					17 - Necesito entre dos y tres días hablando poco o guardando silencio para recuperar mi voz.	T1: Preciso de dois a três dias falando pouco ou mantendo silêncio para recuperar minha voz.	Preciso de dois a três dias falando pouco ou ficando em silêncio para recuperar minha voz.	R1: Necesito de dos a tres días hablando poco o en silencio para recuperar la plenitud de mi voz.	Preciso de dois a três dias falando pouco ou ficando em silêncio para recuperar minha voz.
T2: Preciso de dois a três dias falando pouco ou mantendo-me em silêncio, para recuperar minha voz.	R2: Necesito de dos a tres días hablando poco o guardando silencio para recuperar mi voz.								
T3: Preciso de dois a três dias falando pouco ou ficando em silêncio para recuperar minha voz.									

**Caption**: T1: Spanish-Portuguese translator 1; T2: Spanish-Portuguese translator 2; T3: Spanish-Portuguese translator 3; R1: Portuguese-Spanish back-translator 1; R2: Portuguese-Spanish back-translator 2

In the back-translation stage, disagreement between the two translators occurred exclusively in relation to the scale items. Structural divergences were identified in 14 items (1, 2, 3, 4, 5, 6, 7, 8, 9, 10, 11, 13, 15, and 17), involving differences in sentence structure and wording that did not alter the original meaning. In items 12, 14, and 16, disagreements involved conceptual content, particularly variations in terminology selection and in the expression of the translated concepts.

Overall, teachers assigned satisfactory levels of importance, clarity, and interpretability to most items of the vocal fatigability detection scale. Consequently, the transculturally adapted version of the EFV-D was established (Appendix 1). All data related to each stage of the adaptation process are presented in Chart 1.

Following item evaluation, most scale items (n = 12; 76.6%) were classified by teachers as absolutely relevant. This proportion was significantly higher (p = 0.001) than the proportion of items classified as not relevant (n = 2; 11.7%) or requiring revision (n = 2; 11.7%), as shown in Table 1. Modifications were made to the wording of items 10, 13, 14, and 16 based on the principle of syntactic simplification, aiming to make the language more accessible and comprehensible for teachers. The items considered irrelevant or requiring revision — items 13, 14, 16, and 17 — underwent adjustments intended to improve clarity and interpretation. Nevertheless, these items were retained in the scale because they address relevant aspects associated with vocal fatigability.

**Table 1:** Description of the relevance of the items of the *Escala de Fatigabilidade Vocal em Docentes* (EFV-D) as evaluated by teachers

**Relevance of the items**	**n**	**%**	**Description of the items**
Absolutely relevant item	13 items	76,6	1 to 12 and 15 - Items considered relevant
Item needs changes	2 items	11,7	Item 14 - 34.8% of participants stated that the item needed change. Item 16 - 30.4% of participants stated that the item needed change.
Irrelevant item	2 items	11,7	Item 13 - 30.4% of participants stated that the item is irrelevant. Item 17 - 34.8% of participants stated that the item is irrelevant.
p-value	0,001		

**Relevance level:** relative frequency (%) > 50% for relevance. **Proportion test:** significance.

Finally, considering the need to ensure semantic and contextual equivalence in the Brazilian Portuguese version of the instrument, the expert committee decided to modify the term “docente,” originally present in the title and items of the Spanish version. This term was replaced by “professor” in the Brazilian version. Although both terms are semantically related, the committee considered “professor” to be more appropriate according to cultural equivalence and comprehensibility criteria.

Within the Brazilian context, the term “professor” is more frequently used and more readily recognized by professionals working in elementary, secondary, and higher education. Furthermore, it predominates in official documents, national scientific literature on vocal health^(2-3,9,14^ ), and everyday language. This decision was validated by the expert committee during the final stage of semantic evaluation and was considered more appropriate for preserving the original construct of the scale without compromising its clarity or applicability to the target population. After this final adjustment, the final version of the scale transculturally adapted into Brazilian Portuguese was established (Appendix 1).

## DISCUSSION

​This study aimed to perform the cross-cultural adaptation of the *Escala de Fatigabilidad Vocal en Docentes (EFV-D)* into Brazilian Portuguese to ensure its applicability within the Brazilian context. The analysis of the results obtained throughout the translation, back-translation, expert committee review, and pre-testing stages revealed important linguistic and conceptual adjustments, conducted according to internationally established methodological principles^(12-13)^.

Cross-cultural adaptation represents the initial stage of the validation process and aims to adjust the content of an instrument to ensure its applicability in populations from different linguistic and cultural contexts. Rather than consisting of a literal translation, this process seeks to eliminate sociocultural divergences and to produce a final version that is both appropriate and conceptually equivalent to the original instrument ^(15)^. Therefore, the adaptation process requires consideration of semantic, idiomatic, conceptual, linguistic, experiential, and contextual equivalences related to the adapted construct^(8)^.

The consensus process adopted for both translation and back-translation in the present study was based on the principle of syntactic simplification, involving the reduction of textual complexity without altering content. This strategy aimed to eliminate redundancies, improve message clarity, and facilitate comprehension of the information^(15-16)^, thereby enabling understanding among teachers from different educational levels.

During the initial translation stage, substantial variation was observed among the versions proposed by the translators, particularly in items related to vocal intensity and physical discomfort associated with prolonged voice use. Items 8 and 11 were especially noteworthy because of divergences in the terminology employed, reflecting the inherent complexity involved in translating subjective expressions associated with vocal perception. These discrepancies required specific interventions by the expert committee, which, based on criteria of clarity, semantic relevance, and cultural familiarity, implemented targeted adjustments to preserve the original meaning of the items while facilitating comprehension among Brazilian teachers.

​The decisions made by the expert committee reflected particular attention to the linguistic appropriateness of the scale. Syntactic and lexical modifications were implemented, especially in items 10, 13, 14, and 16, to simplify the wording and improve accessibility. Adjustments to sentence structure and item formulation were prioritized, considering that consistency in wording may influence teachers’ responses by facilitating comprehension and response selection^(17)^. It should be emphasized that items initially considered of limited relevance were retained in the final version because they address important aspects of the vocal fatigability construct, thereby reinforcing the methodological commitment to preserving the conceptual integrity of the instrument.

Items 8 and 11 presented conceptual differences among the translated versions, requiring reformulation during the synthesis stage. In item 8, a duplication of meaning was identified, as the terms “voz irritada” and “garganta irritada” refer to different perceptions. The term “garganta” was retained because it refers to a physical sensation, whereas “voz irritada” is associated with changes in vocal quality. In item 11, discrepancies emerged between the terms “garganta irritada” and “pescoço irritado.” The term “garganta” refers to sensations directly associated with the vocal tract, whereas “pescoço” is more closely related to symptoms resulting from physical effort associated with voice use. Therefore, the term “pescoço” was maintained in the final version.

Regarding the acceptability of the scale items, most teachers considered items 1, 2, 3, 4, 5, 6, 7, 8, 9, 10, 11, 12, and 15 to be highly relevant. These items are predominantly associated with sensations perceived during voice use. The high relevance index attributed to most items by participating teachers reinforces the acceptability and pertinence of the scale content.

Items 13, 14, 16, and 17, although they presented the highest rates of irrelevance or need for revision, were retained in the final version because they address essential aspects for detecting vocal fatigability in teachers and were not considered irrelevant by most evaluators. These items encompass vocal recovery strategies and limitations resulting from excessive voice use, dimensions that may be less immediately perceptible and therefore have been considered less important by some participants.

​Nevertheless, their inclusion is justified because they address fundamental aspects required for understanding vocal fatigability, particularly behaviors adopted in response to prolonged vocal effort and strategies used for vocal recovery. Items 16 and 17, specifically, are considered by the authors of the original study to be essential for quantifying recovery time, an aspect that distinguishes vocal fatigue from vocal fatigability (“16- Preciso dormir uma noite inteira para deixar de sentir desconforto ao falar”; “17- Preciso de dois a três dias falando pouco ou ficando em silêncio para recuperar minha voz”). Considering that vocal fatigability is associated with continuous vocal exposure and insufficient recovery, factors that may contribute over time to the worsening of vocal dysfunctions, the inclusion of these items is justified because they provide relevant information not only for the early identification of fatigue, but also for the planning of therapeutic strategies directed toward teachers’ vocal health.

Another relevant aspect concerns the terminological adjustment related to the designation of the target population of the scale. The replacement of the term “docente” with “professor”was based on the principle of cultural equivalence. Although semantically similar, these terms differ regarding their use and recognition within the Brazilian context, where “professor” is more broadly used across educational levels and more frequently found in the national literature on vocal health. This decision, validated by the expert committee, was considered essential to optimize clarity and facilitate participants’ identification with the purpose of the scale, without compromising the conceptual fidelity of the original version.

Following the completion of the cross-cultural adaptation into Brazilian Portuguese, the EFV-P will undergo the validation process conducted by the authors of the present study to ensure the psychometric properties of validity, reliability, and precision of the adapted version, thereby enabling its application in both research and clinical practice.

The subsequent stages of instrument validation should focus on investigating the validity and reliability properties of the Brazilian Portuguese version of the scale to confirm its robustness for clinical and research applications. This process will enable the use of the EFV-P as an auxiliary tool in speech-language pathology assessment and intervention involving teachers, who constitute a professional group particularly vulnerable to vocal disorders that may negatively impact quality of life, communication, and occupational satisfaction. The identification of vocal fatigability through the EFV-P may enable speech-language pathologists to implement preventive strategies aimed at avoiding progression to more severe dysphonia conditions^(5,6)^.

## CONCLUSION

​The EFV-D was successfully cross-culturally adapted into Brazilian Portuguese, resulting in the version entitled Escala de Fatigabilidade Vocal em Professores (EFV-P). The stages of translation, back-translation, expert committee review, and semantic evaluation ensured the conceptual equivalence and clarity of the items while respecting the linguistic and cultural specificities of the target population. Future studies should investigate the psychometric properties of the adapted version to verify its validity, reliability, and precision.

## Appendix 1 Transculturally adapted version of the EFV-D into Brazilian Portuguese, entitled *Escala de Fatigabilidade Vocal em Professores* (EFV-P)

Escala De Fatigabilidade Vocal Em Professores (EFV-P)

As frases abaixo apresentam alguns sintomas frequentemente associados a problemas de voz. Assinale a resposta que indica o quanto você apresenta o mesmo sintoma

**Table:**

	Nunca	Quase nunca	Às vezes	Quase sempre	Sempre
**Fator 1 - Fadiga no rendimento vocal**					
1. Depois de mais de duas horas falando em forte intensidade, não consigo continuar em tarefas de grande exigência vocal.	0	1	2	3	4
2. Depois de mais de duas horas falando em forte intensidade, sinto a voz cansada.	0	1	2	3	4
3. Depois de mais de duas horas falando em forte intensidade, percebo uma crescente sensação de esforço vocal.	0	1	2	3	4
4. Depois de mais de 2 horas falando em forte intensidade, minha voz fica rouca.	0	1	2	3	4
5. Depois de mais de 2 horas falando em forte intensidade, tenho que fazer esforço para continuar falando como estava fazendo.	0	1	2	3	4
6. Depois de mais de duas horas falando em forte intensidade, sinto dificuldade para continuar projetando a voz.	0	1	2	3	4
7. Depois de mais de duas horas falando em forte intensidade, sinto minha voz fraca.	0	1	2	3	4
8. Depois de mais de 2 horas falando em forte intensidade, sinto a garganta irritada.	0	1	2	3	4
9. Depois de mais de 2 horas falando em forte intensidade, sinto desconforto no pescoço, na garganta ou na língua.	0	1	2	3	4
10. Depois de mais de duas horas falando em forte intensidade, sinto dificuldade para falar baixo.	0	1	2	3	4
11. Depois de um dia inteiro usando a voz, sinto desconforto ou dor no pescoço.	0	1	2	3	4
**Fator 2 - Condutas reparadoras**					
12. Depois de um tempo utilizando a voz, costumo limitar a quantidade de fala.	0	1	2	3	4
13. Evito situações sociais em que sei que vou falar muito.	0	1	2	3	4
14. Sinto que não consigo falar com minha família depois de um dia de trabalho.	0	1	2	3	4
15. Preciso ficar em silêncio e me hidratar por mais de uma hora para deixar de sentir esforço para falar.	0	1	2	3	4
16. Preciso dormir uma noite inteira para deixar de sentir desconforto ao falar.	0	1	2	3	4
17. Preciso de dois a três dias falando pouco ou ficando em silêncio para recuperar minha voz.	0	1	2	3	4
