# ERRATUM: Cross-cultural adaptation of the *Escala De Fatigabilidad Vocal en Docentes (EFV-D)* to brazilian portuguese

**DOI:** 10.1590/2317-1782/e20260189en

**Published:** 2026-09-25

Due to an honest mistake by the authors, the article “Cross-cultural adaptation of the *Escala De Fatigabilidad Vocal en Docentes (EFV-D)* to brazilian portuguese” (DOI https://doi.org/10.1590/2317-1782/e20250426en), present in CoDAS 2026;38(5):e20250426, was published without identifying all authors and with an incorrect authorship order.

**Where it was previously identified:**

Maria Eduarda Oliveira de Figueiredo, Priscila Oliveira, Thays Vaiano, Thiago Henrique de Pontes Ferreira and Larissa Nadjara Alves Almeida.

**Now identify:**

Maria Eduarda Oliveira de Figueiredo, Larissa Nadjara Alves Almeida, Thays Vaiano, Thiago Henrique de Pontes Ferreira, Silvia Contreras-Regatero and Priscila Oliveira.

The authors apologize for the error.

**Silvia Contreras-Regatero**

**ORCID:** https://orcid.org/0000-0002-1589-4903

**Affiliation:** Department of psychology and speech language pathology, Universitat Ramon Llull, Barcelona, Espanha.

**Contribution:** Validation and Writing – review and editing.

Editor:

Aline Mansueto Mourão

