# Supplementary material for: Cross-cultural adaptation of the Escala De Fatigabilidad Vocal en Docentes (EFV-D) to brazilian portuguese
Source: Codas. 2026 Aug 17;38(5):e20250426. doi: 10.1590/2317-1782/e20250426en (PMC13520641; doi:10.1590/2317-1782/e20250426en)
Supplement: Translation

# Translation: Adaptação transcultural da *Escala de Fatigabilidad Vocal en Docentes (EFV-D)* para o português brasileiro

**DOI:** 10.1590/2317-1782/e20250426pt

**Authors:** Maria Eduarda Oliveira de Figueiredo, Priscila Oliveira, Thays Vaiano, Thiago Henrique de Pontes Ferreira, Larissa Nadjara Alves Almeida

**Affiliations:** 1 Departamento de Fonoaudiologia, Universidade Federal da Paraíba – UFPB - João Pessoa (PB), Brasil.; 2 Centro de Estudos da Voz – CEV - São Paulo (SP), Brasil.; 3 Faculdade IDE – Sergipe (SE), Brasil.

**Keywords:** Voz, Distúrbios da Voz, Fadiga, Docentes, Autoavaliação Diagnóstica, Estudo de Validação

## Abstract

**Objetivo:**

Adaptar transculturalmente a Escala de Fatigabilidad Vocal en Docentes (EFV-D) para o português brasileiro (PB).

**Método:**

Os procedimentos seguiram as recomendações de Beaton et al. e as diretrizes da International Test Commission (ITC). Desenvolvida e validada em espanhol, a EFV-D detecta a fatigabilidade vocal em professores. É composta por 17 itens divididos em dois fatores: 11 relacionados às sensações durante o uso da voz e seis às estratégias de recuperação e limitações devido ao uso vocal excessivo. O processo incluiu cinco etapas: (1) tradução para o PB por dois fonoaudiólogos e um tradutor juramentado, brasileiros e fluentes em espanhol; (2) síntese das traduções; (3) pré-teste com 23 professores do ensino fundamental, médio e superior, que avaliaram a relevância dos itens; (4) retrotradução realizada por um fonoaudiólogo e um tradutor não especialista; e (5) consenso das versões e elaboração da versão final pelo comitê de especialistas.

**Resultados:**

Na tradução, houve ajustes no título do fator 2 e em todos os itens, sendo 15 reformulados e dois com conteúdo alterado. Na retrotradução, observaram-se divergências estruturais em 14 itens e de conteúdo em 3, solucionadas pelo comitê. De modo geral, os professores atribuíram níveis satisfatórios de importância, clareza e interpretação à maioria dos itens. Aqueles com relevância inferior a 50% foram modificados conforme os critérios de equivalência.

**Conclusão:**

A adaptação transcultural da EFV-D para o PB resultou na versão final denominada Escala de Fatigabilidade Vocal em Professores (EFV-P). Estudos futuros devem ser realizados para verificar sua validade e a confiabilidade.

## INTRODUÇÃO

Os professores representam a categoria de profissionais da voz mais acometida por distúrbios vocais, pois utilizam a voz como principal ferramenta de trabalho, o que os torna suscetíveis ao desenvolvimento de disfonias^(1)^. As condições ambientais e organizacionais do trabalho docente, como ruído, poeira, jornadas prolongadas e excesso de tarefas, contribuem para o comprometimento da saúde vocal. A carga horária excessiva impõe maior exigência fonatória e sobrecarga do aparelho fonador, favorecendo o surgimento de sintomas e alterações vocais^(2)^. Entre os sintomas vocais mais relatados por esses profissionais, destacam-se: tensão muscular, esforço ao falar, redução da projeção, rouquidão, e fadiga, que é a sensação física mais recorrente^(3)^.

A fadiga vocal é caracterizada por sintomas autopercebidos, que se manifestam por meio do aumento no esforço fonatório em resposta à elevação da demanda vocal. Esses sintomas tendem a melhorar com o repouso adequado da voz. Atualmente, a definição de fadiga vocal envolve duas grandes manifestações: a fadiga de desempenho e a fadiga percebida^(4,5)^.

Na fadiga de desempenho, há um declínio na qualidade vocal ao longo de um período específico, que está relacionado a três fatores principais: as propriedades viscoelásticas das pregas vocais, a capacidade contrátil dos músculos envolvidos, e a habilidade do sistema nervoso de fornecer sinais de ativação adequados, conforme as vias aferentes e eferentes que regulam a tarefa vocal^(4,5)^.

Em contrapartida, a fadiga percebida pode se manifestar durante ou após a atividade de fala. Essa percepção é influenciada por moduladores como o humor, a motivação, a dor e o feedback do próprio indivíduo, atuando como um sinal de que é necessário realizar uma pausa na utilização da voz^(4,5)^.

A fadiga vocal ocasional é capaz de causar danos isolados ao sistema fonatório espontaneamente recuperáveis com o repouso adequado. No entanto, a fadiga vocal recorrente, denominada por Contreras-Regatero e Vila-Rovira (2024) como “fatigabilidade” corresponde à sensação generalizada de fadiga mantida ao longo de períodos prolongados, resultante da interação entre a fadiga de desempenho e a fadiga percebida. Nesse quadro, que ultrapassa episódios isolados e pontuais, ocorre dano contínuo e o processo de cicatrização permanece em um estado perpétuo de reparo, podendo não alcançar a recuperação completa^(5,6)^. Por se tratar de um parâmetro mais abrangente, permite a compreensão dos impactos da fadiga na participação em atividades cotidianas e na qualidade de vida do indivíduo^(5)^.

Para avaliar e mensurar a fatigabilidade vocal é necessário entender a autopercepção do paciente sobre esse aspecto. Isso é possível por meio da autoavaliação vocal, uma das etapas primordiais na avaliação multidimensional da voz. A autoavaliação vocal é realizada por meio de instrumentos, compostos por itens, que permitem a coleta de informações essenciais para o diagnóstico vocal, aspectos que não são identificados por outros métodos avaliativos. A ausência da perspectiva do paciente sobre sua voz torna a avaliação incompleta^(7)^.

Os instrumentos de autoavaliação devem ser adequados ao constructo e à população que se deseja avaliar, de modo que suas interpretações produzam resultados válidos, mensurando, de fato, aquilo a que se propõem. Além disso, devem apresentar estabilidade, precisão e equidade, analisando os indivíduos de forma imparcial^(8)^.

Existem instrumentos direcionados aos professores, como o Condição de Produção Vocal do Professor (CPV-P), que caracteriza o perfil vocal e as condições laborais às quais estão expostos, o Índice de Triagem de Distúrbio de Voz (ITDV), utilizado para identificar indivíduos com risco de apresentar alterações vocais^(9,10)^, e o Índice de Fadiga Vocal (IFV) que auxilia na identificação da fadiga vocal, mas não possui especificidade para relatar as sensações vocais relacionando-as à atividade vocal exigente que as desencadeou^(11)^. Dessa forma, é importante destacar que, embora esses instrumentos sejam direcionados a professores, eles não avaliam a fatigabilidade vocal. A *Escala De Fatigabilidad Vocal En Docentes* (*EFV-D*), por sua vez, investiga a relação entre a fatigabilidade vocal em professores e sua qualidade de vida, buscando associar as sensações vocais às demandas que as desencadearam^(5,6)^.

Considerando que a fatigabilidade vocal é um sintoma frequente na população docente, torna-se essencial a utilização de um instrumento específico e sensível às suas especificidades, como a EFV-D*.* Desenvolvida e validada em espanhol, na cidade de Barcelona, a escala tem como objetivo detectar a fatigabilidade vocal em professores. É composta por 17 itens, divididos em dois fatores: onze relacionados às sensações durante o uso da voz e seis associados às estratégias de recuperação e às limitações devido ao uso vocal excessivo^(6)^.

A investigação da fatigabilidade vocal em professores brasileiros por meio da EFV-D será viabilizada a partir de sua adaptação transcultural e validação para a cultura brasileira, o que permitirá a obtenção de dados relevantes para a avaliação e a intervenção fonoaudiológica. O artigo que apresentou o processo de validação da escala em sua versão original incluiu uma tradução livre para o português brasileiro, mas sem o cumprimento de nenhuma das etapas de validação de conteúdo recomendadas por diretrizes internacionais. Dessa forma, este estudo tem como objetivo realizar a adaptação transcultural da EFV-D para o português brasileiro (PB).

## MÉTODO

Trata-se de um estudo de delineamento transversal e natureza metodológica, aprovado pelo Comitê de Ética em Pesquisa, sob parecer 7.406.255. A pesquisa seguiu as orientações propostas pela Resolução CNS 466/12, e todos os participantes assinaram o Termo de Consentimento Livre Esclarecido (TCLE).

O presente estudo teve como foco a adaptação transcultural da *Escala De Fatigabilidad Vocal En Docentes (EFV-D)*, voltada à população docente. O objetivo foi adaptar a escala linguística e culturalmente, assegurando sua equivalência conceitual e adequação ao contexto sociocultural, de modo a viabilizar sua aplicação como instrumento sensível e específico na detecção da fatigabilidade vocal em professores brasileiros. Os procedimentos da adaptação transcultural da EFV-D para o PB seguiram as recomendações de Beaton et al.^(12)^ e as diretrizes da *International Test Commission* (ITC)^(13)^.

A EFV-D foi desenvolvida e validada em espanhol, na cidade de Barcelona. A escala tem como objetivo avaliar os níveis de fadiga vocal dos professores e analisar variações com base em características pessoais e ocupacionais. É composta por 17 itens agrupados em dois fatores: Fator 1 - Fadiga no desempenho vocal, composto por 11 itens (itens 1 a 11), avalia as sensações e percepções relacionadas à fadiga durante o uso da voz; e Fator 2 - Condutas reparadoras, com 6 itens (itens 12 a 17), investiga as estratégias utilizadas para recuperar a voz. Cada item possui uma chave de resposta com escala Likert de cinco pontos: 0 = Nunca; 1 = Quase nunca; 2 = Às vezes; 3 = Quase sempre; 4 = Sempre. O escore é obtido por meio da somatória simples das respostas de todos os itens. A classificação do nível de fadiga dos professores ocorre em três níveis, de acordo com os pontos de corte: sem fatigabilidade (pontuação <15), fatigabilidade moderada (pontuação entre 15-27) e fatigabilidade alta (pontuação >28)^(6)^.

O processo de adaptação transcultural para o PB incluiu cinco etapas: 1) tradução para o português brasileiro, 2) síntese das traduções, 3) pré-teste, 4) retrotradução, 5) consenso das versões retrotraduzidas e elaboração da versão final. Todas as etapas foram conduzidas de forma online e, para a adaptação transcultural, foram considerados o título, os fatores e todos os itens da escala.

A tradução (etapa 1) foi realizada de forma independente por três tradutores, sendo dois fonoaudiólogos e um tradutor juramentado, brasileiros e fluentes em espanhol, que receberam a versão em espanhol e a traduziram com base na equivalência cultural. O comitê de especialistas, composto pelos autores da pesquisa, elaborou em consenso uma síntese das traduções (etapa 2) que resultou em uma versão única a partir das três traduções do título, dos fatores e dos itens. Essa versão foi submetida à etapa de pré-teste (etapa 3) para uma avaliação preliminar da compreensão e aplicabilidade da escala por professores em atuação profissional no momento da pesquisa.

Os professores participantes desta etapa foram recrutados virtualmente por meio de um convite contendo o link de acesso à plataforma *Google Forms*. No formulário online, tiveram acesso ao TCLE, à versão transculturalmente adaptada da escala e às instruções de resposta, devendo avaliar os itens com base nas seguintes opções: 1 = Item não relevante; 2 = Item que necessita de revisão para avaliação da relevância; 3 = Item relevante, mas que requer pequenas alterações; 4 = Item absolutamente relevante. Os professores foram orientados a sugerir modificações na redação dos itens considerados inadequados à cultura ou de difícil compreensão. Após a coleta, os dados foram tabulados em uma planilha, realizada a análise de frequência relativa e os itens que obtiveram índice de relevância igual ou inferior a 50% foram revisados. As sugestões apontadas pelos participantes foram analisadas e acatadas.

A amostra foi composta por 23 professores, sendo 6 do ensino fundamental (26,1%), 9 do ensino médio (39,1%) e 8 do ensino superior (34,8%). As idades variaram entre 23 e 65 anos, com média de 39 anos e 7 meses (DP = 13,48). Os critérios de elegibilidade foram: ser professor com idade igual ou superior a 18 anos, falantes nativos do português brasileiro e atuar no ensino fundamental, médio ou superior.

A versão estabelecida após a realização do pré-teste foi submetida à retrotradução (etapa 4) para o idioma de origem por dois tradutores fluentes em espanhol e falantes nativos do PB, sendo um fonoaudiólogo e um professor de espanhol, com o objetivo de avaliar possíveis alterações no conteúdo original. O comitê elaborou, por consenso, uma síntese das versões retrotraduzidas, a qual foi posteriormente comparada e analisada em relação à versão original da EFV-D, considerando as equivalências semânticas, idiomáticas, conceituais, linguísticas, experienciais e contextuais (etapa 5). A partir dessa análise, foi estabelecida a versão final da escala, transculturalmente adaptada para o PB (Apêndice 1).

Os dados referentes à avaliação dos professores sobre a relevância dos itens da escala então denominada Escala de Fatigabilidade Vocal em Professores (EFV-P) foram analisados de forma quantitativa por meio de análise descritiva, medidas de frequências absolutas e relativas, e inferencial, através do teste de comparação de proporções. As respostas foram classificadas como “Item não relevante”, “Item que necessita de revisão para avaliação da relevância” e “Item relevante, mas que requer pequenas alterações”, e “Item completamente relevante”, conforme as opções assinaladas na chave de resposta. Para tais análises, utilizou-se o software *Statistical Package for the Social Sciences* (SPSS), versão 25.0.0, adotando nível de significância de 5% (p< 0,05).

## RESULTADOS

Na etapa de tradução, houve concordância quanto ao nome do instrumento: Escala de Fatigabilidade Vocal em Docentes (EFV-D) e ao título do fator 1: Fadiga no rendimento vocal. No entanto, no fator 2, houve discordância entre os três tradutores, sendo necessário o ajuste pelo comitê de especialistas para “Condutas reparadoras”.

Na tradução dos itens, os tradutores propuseram escritas diferentes em 15 afirmativas, sendo elas: 1, 2, 3, 4, 5, 6, 7, 9, 10, 12, 13, 14, 15, 16 e 17. Nos itens 8 e 11, houve discordância quanto ao conteúdo. No item 8, as opções propostas foram: “Depois de mais de 2 horas falando em forte intensidade, sinto minha voz irritada”; “Depois de mais de 2 horas falando em alta intensidade, sinto a garganta irritada”; e “Depois de mais de 2 horas falando em alta intensidade, sinto a voz irritada”. No item 11, as versões sugeridas foram: “Sinto desconforto ou dor no pescoço depois de um dia inteiro usando a voz”; “No final do dia, sinto incômodo ou dor na garganta usando minha voz”; e “Sinto desconforto ou dor no pescoço ao final de um dia usando a voz”. De modo geral, observou-se inconsistência entre os termos “forte” e “alta” ao se referirem à intensidade vocal.

Ao sintetizar as versões traduzidas, o comitê de especialistas manteve os itens 1, 2, 3, 7, 11, 13, 15, 16 e 17 conforme pelo menos uma das traduções realizadas. Já os itens 4, 5, 6, 8, 9, 10, 12 e 14 foram modificados com o objetivo de tornar a linguagem mais adequada e compreensível à cultura e população-alvo (Quadro 1). Foram realizados ajustes na redação dos itens que apresentaram índice de relevância igual ou inferior a 50%, atendendo aos critérios de equivalência e considerando as sugestões propostas pelos professores. Optou-se por manter o termo "forte" ao se referir à intensidade vocal.

**Quadro 1:** Resultado do processo de adaptação transcultural da EFV-D para o português brasileiro

**Versão original**	**Tradução**	**Síntese**	**Retrotradução**	**Versão final**
**Título**				
Escala de Fatigabilidad Vocal en Docentes (EFV-D)	T1: Escala de Fatigabilidade Vocal em Docentes (EFV-D)	Escala de Fatigabilidade Vocal em Docentes (EFV-D)	R1: Escala de Fatigabilidad Vocal en Docentes (EFV-D)	Escala de Fatigabilidade Vocal em Docentes (EFV-D)
	T2: Escala de Fatigabilidade Vocal em Docentes (EFV-D)			
			R2: Escala de Fatigabilidad Vocal en Docentes (EFV-D)	
	T3: Escala de Fatigabilidade Vocal em Docentes (EFV-D)			
**Fator 1**				
Fatiga en el rendimiento vocal	T1: Fadiga no rendimento vocal	Fadiga no rendimento vocal	R1: Fatiga en el rendimiento vocal	Fadiga no rendimento vocal
	T2: Fadiga no rendimento vocal			
			R2: Fatiga en el rendimiento vocal	
	T3: Fadiga no rendimento vocal			
**Fator 2**				
Conductas reparadoras	T1: Condutas reparadoras	Condutas reparadoras	R1: Conductas reparadoras	Condutas reparadoras
	T2: Hábitos de recuperação vocal			
			R2: Conductas reparadoras	
	T3: Comportamentos reparadores			
**Itens**				
1 - Después de más de 2 horas hablando a una intensidad alta no puedo seguir con tareas vocales exigentes.	T1: Depois de mais de 2 horas falando em forte intensidade, não consigo continuar em tarefas de grande exigência vocal.	Depois de mais de 2 horas falando em forte intensidade, não consigo continuar em tarefas de grande exigência vocal.	R1: Después de más de 2 horas hablando con alta intensidad, no puedo continuar con tareas que exigen mucho esfuerzo vocal.	Depois de mais de 2 horas falando em forte intensidade, não consigo continuar em tarefas de grande exigência vocal.
	T2: Depois de mais de 2 horas falando em alta intensidade, não posso continuar com tarefas vocais intensas.			
			R2: Después de más de 2 horas hablando con fuerte intensidad, no puedo seguir las tareas de gran exigencia vocal.	
	T3: Depois de mais de 2 horas falando em alta intensidade, não consigo continuar com tarefas vocais exigentes.			
2 - Después de más de 2 horas hablando a una intensidad alta siento la voz cansada.	T1: Depois de mais de 2 horas falando em forte intensidade, sinto a voz cansada.	Depois de mais de 2 horas falando em forte intensidade, sinto a voz cansada.	R1: Después de más de 2 horas hablando con alta intensidad, siento la voz cansada.	Depois de mais de 2 horas falando em forte intensidade, sinto a voz cansada.
	T2: Depois de mais de 2 horas falando em alta intensidade, sinto a voz cansada.			
			R2: Después de más de 2 horas hablando con fuerte intensidad, siento la voz cansada.	
	T3: Depois de mais de 2 horas falando em alta intensidade, sinto a voz cansada.			
3 - Después de más de 2 horas hablando a una intensidad alta experimento una creciente sensación de esfuerzo en la voz.	T1: Depois de mais de 2 horas falando em forte intensidade, percebo uma crescente sensação de esforço vocal.	Depois de mais de 2 horas falando em forte intensidade, percebo uma crescente sensação de esforço vocal.	R1: Después de más de 2 horas hablando con alta intensidad, noto una creciente sensación de esfuerzo vocal.	Depois de mais de 2 horas falando em forte intensidade, percebo uma crescente sensação de esforço vocal.
	T2: Depois de mais de 2 horas falando em alta intensidade, sinto uma crescente sensação de esforço na minha voz.			
			R2: Después de más de 2 horas hablando con fuerte intensidad, me doy cuenta de una creciente sensación de esfuerzo vocal.	
	T3: Depois de mais de 2 horas falando em alta intensidade, experimento uma sensação crescente de esforço na voz.			
4 - Después de más de 2 horas hablando a una intensidad alta mi voz se vuelve ronca.	T1: Depois de mais de 2 horas falando em forte intensidade, fico com a voz rouca.	Depois de mais de 2 horas falando em forte intensidade minha voz fica rouca.	R1: Después de más de 2 horas hablando con alta intensidad, mi voz se vuelve ronca.	Depois de mais de 2 horas falando em forte intensidade, minha voz fica rouca.
	T2: Depois de mais de 2 horas falando em alta intensidade minha voz fica rouca.			
			R2: Después de más de 2 horas hablando con fuerte intensidad, mi voz se vuelve ronca.	
	T3: Depois de mais de 2 horas falando em alta intensidade, minha voz fica rouca.			
5 - Después de más de 2 horas hablando a una intensidad alta me resulta un esfuerzo seguir hablando como lo estaba haciendo.	T1: Depois de mais de 2 horas falando em forte intensidade, tenho que fazer esforço para seguir falando como antes.	Depois de mais de 2 horas falando em forte intensidade, tenho que fazer esforço para continuar falando como estava fazendo.	R1: Después de más de 2 horas hablando con alta intensidad, tengo que hacer un esfuerzo para seguir hablando como lo estaba haciendo.	Depois de mais de 2 horas falando em forte intensidade, tenho que fazer esforço para continuar falando como estava fazendo.
	T2: Depois de mais de 2 horas falando em alta intensidade é um esforço para continuar falando como eu estava fazendo.			
			R2: Después de más de 2 horas hablando con fuerte intensidad, tengo que esforzarme para continuar hablando como lo estaba haciendo.	
	T3: Depois de mais de 2 horas falando em alta intensidade, é um esforço continuar falando como estava.			
6 - Después de más de 2 horas hablando a una intensidad alta me resulta difícil seguir proyectando la voz.	T1: Depois de mais de 2 horas falando em forte intensidade, sinto dificuldade para projetar a voz.	Depois de mais de 2 horas falando em forte intensidade, sinto dificuldade para continuar projetando a voz.	R1: Después de más de 2 horas hablando con alta intensidad, siento dificultad para seguir proyectando la voz.	Depois de mais de 2 horas falando em forte intensidade, sinto dificuldade para continuar projetando a voz.
	T2: Depois de mais de 2 horas falando em alta intensidade, torna-se difícil continuar projetando a voz.			
			R2: Después de más de 2 horas hablando con fuerte intensidad, siento dificultad para seguir proyectando la voz.	
	T3: Depois de mais de 2 horas falando em alta intensidade, é difícil continuar projetando a voz.			
7 - Después de más de 2 horas hablando a una intensidad alta siento mi voz débil.	T1: Depois de mais de 2 horas falando em forte intensidade, sinto minha voz fraca.	Depois de mais de 2 horas falando em forte intensidade, sinto minha voz fraca.	R1: Después de más de 2 horas hablando con alta intensidad, siento mi voz débil.	Depois de mais de 2 horas falando em forte intensidade, sinto minha voz fraca.
	T2: Depois de mais de 2 horas falando em alta intensidade, sinto minha voz fraca.			
			R2: Después de más de 2 horas hablando con fuerte intensidad, siento mi voz débil.	
	T3: Depois de mais de 2 horas falando em alta intensidade, sinto minha voz fraca.			
8 - Después de más de 2 horas hablando a una intensidad alta siento la voz irritada.	T1: Depois de mais de 2 horas falando em forte intensidade, sinto minha voz irritada.	Depois de mais de 2 horas falando em forte intensidade, sinto a garganta irritada.	R1: Después de más de 2 horas hablando con alta intensidad, siento dolor de garganta.	Depois de mais de 2 horas falando em forte intensidade, sinto a garganta irritada.
	T2: Depois de mais de 2 horas falando em alta intensidade, sinto a garganta irritada.			
			R2: Después de más de 2 horas hablando con fuerte intensidad, siento la garganta irritada.	
	T3: Depois de mais de 2 horas falando em alta intensidade, sinto a voz irritada.			
9 - Después de más de 2 horas hablando a una intensidad alta siento incomodidad en el cuello, la garganta o la lengua.	T1: Depois de mais de 2 horas falando em forte intensidade, sinto incomodo no pescoço, garganta ou língua.	Depois de mais de 2 horas falando em forte intensidade, sinto desconforto no pescoço, na garganta ou na língua.	R1: Después de más de 2 horas hablando con alta intensidad, siento malestar en el cuello, la garganta o la lengua.	Depois de mais de 2 horas falando em forte intensidade, sinto desconforto no pescoço, na garganta ou na língua.
	T2: Depois de mais de 2 horas falando em alta intensidade sinto desconforto no pescoço, na garganta ou na língua.			
			R2: Después de más de 2 horas hablando con fuerte intensidad, siento molestia en el cuello, en la garganta y en la lengua.	
	T3: Depois de mais de 2 horas falando em alta intensidade, sinto desconforto no pescoço, na garganta ou na língua.			
10 - Después de más de 2 horas hablando a una intensidad alta siento dificultad para hablar con voz suave.	T1: Depois de mais de 2 horas falando em forte intensidade, sinto dificuldade para falar baixo.	Depois de mais de 2 horas falando em forte intensidade, sinto dificuldade para falar baixo/suave.	R1: Después de más de 2 horas hablando con alta intensidad, me cuesta hablar en voz baja.	Depois de mais de 2 horas falando em forte intensidade, sinto dificuldade para falar baixo.
	T2: Depois de mais de 2 horas falando em alta intensidade, sinto dificuldade em falar com uma voz suave.			
			R2: Después de más de 2 horas hablando con fuerte intensidad, siento dificultad para hablar en voz baja.	
	T3: Depois de mais de 2 horas falando em alta intensidade, tenho dificuldade em falar com voz suave.			
11 - Siento malestar o dolor en el cuello al final de un día utilizando la voz.	T1: Sinto desconforto ou dor no pescoço depois de um dia inteiro usando a voz.	Depois de um dia inteiro usando a voz, sinto desconforto ou dor no pescoço.	R1: Después de un día entero usando la voz, siento malestar vocal o dolor en el cuello.	Depois de um dia inteiro usando a voz, sinto desconforto ou dor no pescoço.
	T2: No final do dia, sinto incômodo ou dor na garganta usando minha voz.			
			R2: Después de un día entero usando la voz, siento malestias o dolor en el cuello.	
	T3: Sinto desconforto ou dor no pescoço ao final de um dia usando a voz.			
12 - Generalmente tiendo a limitar el habla después de un periodo de tiempo usando la voz.	T1: Geralmente limito a quantidade de fala depois de um tempo utilizando a voz.	Depois de um tempo utilizando a voz, costumo limitar a quantidade de fala.	R1: Tras un período de uso continuo de la voz, reduzco mi habla para evitar fatiga.	Depois de um tempo utilizando a voz, costumo limitar a quantidade de fala.
	T2: Geralmente costumo limitar a fala depois de um período de tempo considerável usando a voz.			
			R2: Después de un tiempo usando la voz, tiendo a limitar el uso del habla.	
	T3: Geralmente, tenho a tendência de limitar a fala após um período utilizando a voz.			
13 - Evito situaciones sociales en las que sé que tengo que hablar más.	T1: Evito situações sociais em que sei que preciso falar mais.	Evito situações sociais nas quais eu sei que vou falar muito.	R1: Evito situaciones sociales en las que sé que tendré que hablar mucho.	Evito situações sociais em que sei que vou falar muito.
	T2: Evito situações sociais nas quais eu sei que vou falar muito.			
			R2: Evito situaciones sociales en las que sé que voy a hablar mucho.	
	T3: Evito situações sociais nas quais sei que precisarei falar mais.			
14 - Siento que no puedo hablar con mi familia después de un día de trabajo.	T1: Sinto que não consigo falar com minha família depois de um dia de trabalho.	Depois de um dia de trabalho, sinto que não consigo falar com minha família.	R1: Al terminar mi jornada laboral, no tengo energía para hablar con mi familia.	Sinto que não consigo falar com minha família depois de um dia de trabalho.
	T2: Sinto que não posso falar com minha família depois de um dia de trabalho.			
			R2: Siento que no puedo hablar con mi familia después de un día de trabajo.	
	T3: Sinto que não consigo falar com minha família após um dia de trabalho.			
15 - Necesito guardar silencio e hidratarme más de 1 h para dejar de sentir sobreesfuerzo al hablar.	T1: Preciso ficar em silêncio e me hidratar por mais de 1 hora para deixar de sentir esforço para falar.	Preciso ficar em silêncio e me hidratar por mais de 1 hora para deixar de sentir esforço para falar.	R1: Necesito quedarme en silencio e hidratarme por más de 1 hora para dejar de sentir esfuerzo al hablar.	Preciso ficar em silêncio e me hidratar por mais de 1 hora para deixar de sentir esforço para falar.
	T2: Preciso ficar em silêncio e me hidratar por mais de 1 hora para parar de sentir esforço excessivo ao falar.			
			R2: Necesito estar en silencio e hidratarme durante más de 1 hora para dejar de sentir el esfuerzo de hablar.	
	T3: Preciso ficar em silêncio e me hidratar por mais de 1 hora para deixar de sentir sobrecarga ao falar.			
16 - Necesito dormir toda una noche para dejar de sentir malestar al hablar.	T1: Preciso dormir uma noite inteira para deixar de sentir desconforto ao falar.	Preciso dormir uma noite inteira para parar de sentir desconforto ao falar.	R1: Después de sentir malestar al hablar, necesito dormir una noche entera para recuperar la plenitud de mi voz.	Preciso dormir uma noite inteira para deixar de sentir desconforto ao falar.
	T2: Preciso dormir a noite toda para parar de sentir desconforto ao falar.			
			R2: Necesito dormir una noche entera para dejar de sentir malestar al hablar.	
	T3: Preciso dormir uma noite inteira para parar de sentir desconforto ao falar.			
					17 - Necesito entre dos y tres días hablando poco o guardando silencio para recuperar mi voz.	T1: Preciso de dois a três dias falando pouco ou mantendo silêncio para recuperar minha voz.	Preciso de dois a três dias falando pouco ou ficando em silêncio para recuperar minha voz.	R1: Necesito de dos a tres días hablando poco o en silencio para recuperar la plenitud de mi voz.	Preciso de dois a três dias falando pouco ou ficando em silêncio para recuperar minha voz.
T2: Preciso de dois a três dias falando pouco ou mantendo-me em silêncio, para recuperar minha voz.	R2: Necesito de dos a tres días hablando poco o guardando silencio para recuperar mi voz.								
T3: Preciso de dois a três dias falando pouco ou ficando em silêncio para recuperar minha voz.									

**Legenda:** T1: tradutor 1 espanhol-português; T2: tradutor 2 espanhol-português; T3: tradutor 3 espanhol-português; R1: retrotradutor 1 português-espanhol; R2: retrotradutor 2 português-espanhol

Na retrotradução, houve discordância entre os dois tradutores apenas em relação aos itens da escala. Foram identificadas divergências na estrutura das frases em 14 itens: 1, 2, 3, 4, 5, 6, 7, 8, 9, 10, 11, 13, 15 e 17, relacionadas à forma de redação, sem prejuízo do significado original. Nos itens 12, 14 e 16, as discordâncias relacionadas ao conteúdo envolveram variações na escolha dos termos e na expressão do conceito traduzido.

De modo geral, os professores atribuíram à maioria dos itens níveis satisfatórios de importância, clareza e interpretação para compor a escala de detecção da fatigabilidade vocal. Assim, foi estabelecida a versão transculturalmente adaptada da EFV-D (Apêndice 1). Todos os dados referentes a cada etapa estão descritos no Quadro 1.

Após a avaliação, observou-se que a maioria dos itens da escala (n = 12; 76,6%) foi considerada absolutamente relevante pelos professores, sendo esse valor significativamente maior (p = 0,001) em comparação aos itens avaliados como não relevantes (n = 2; 11,7%) ou que necessitaram de revisão (n = 2; 11,7%), conforme apresentado na Tabela 1. Foram realizadas alterações na redação dos itens 10, 13, 14 e 16, com base no princípio da simplificação sintática, a fim de tornar a linguagem mais acessível e compreensível para os professores. Os itens considerados irrelevantes e que precisam de alterações - 13, 14, 16 e 17 - passaram por ajustes com o objetivo de melhorar a clareza e a interpretação. Ainda assim, optou-se por mantê-los na escala, por abordarem aspectos relevantes relacionados à fatigabilidade vocal.

**Tabela 1:** Descrição da relevância dos itens da Escala de Fatigabilidade Vocal em Professores (EFV-P) avaliada pelos professores

**Relevância dos itens**	**n**	**%**	**Descrição dos itens**
Item absolutamente relevante	13 itens	76,6	1 à 12 e 15 - Itens considerados relevantes
Item precisa de alterações	2 itens	11,7	Item 14 - 34,8% dos participantes afirmou que o item precisava de alteração Item 16 - 30,48% dos participantes afirmou que o item precisava de alteração
Item não relevante	2 itens	11,7	Item 13 - 30,48% dos participantes afirmou que o item é irrelevante Item 17 - 34,8% dos participantes afirmou que o item é irrelevante
p-valor	0,001		

Nível de relevância: frequência relativa (%) > 50% para relevância. Teste de proporções: significância.

Por fim, considerando ainda a necessidade de se observar equivalências semânticas e contextuais da versão brasileira do instrumento, o comitê optou por realizar um ajuste quanto à palavra “docente”, presente no título e nos itens da versão original em espanhol. Foi realizada a substituição desse termo pelo termo “professor”, na versão brasileira. O comitê considerou que, embora ambos os termos sejam semanticamente próximos, a palavra “professor” parece ser mais adequada, de acordo com critérios de equivalência cultural e compreensibilidade.

No contexto brasileiro, o termo “professor” é mais amplamente utilizado e reconhecido por profissionais da educação básica e superior, sendo predominante em documentos oficiais, literatura científica nacional sobre saúde vocal^(2,3,9,14)^ e no vocabulário cotidiano. Essa escolha foi validada pelo comitê de especialistas durante a etapa final de avaliação semântica, sendo considerada mais adequada para preservar o constructo original da escala sem comprometer sua clareza ou aplicabilidade no público-alvo. Após esse ajuste final, foi estabelecida a versão final da escala, transculturalmente adaptada para o PB (Apêndice 1).

## DISCUSSÃO

O presente estudo teve como objetivo adaptar transculturalmente a *Escala de Fatigabilidad Vocal en Docentes (EFV-D)* para o português brasileiro, visando garantir sua aplicabilidade no contexto nacional. A análise dos resultados obtidos ao longo do processo de tradução, retrotradução, avaliação por comitê de especialistas e pré-teste evidenciou importantes ajustes linguísticos e conceituais, conduzidos com base em princípios metodológicos internacionalmente estabelecidos^(12,13)^.

A adaptação transcultural constitui a etapa inicial do processo de validação, com o objetivo de ajustar o conteúdo do instrumento para garantir sua aplicabilidade em uma população de outro idioma ou cultura. Busca eliminar divergências socioculturais, não se tratando apenas de uma tradução literal, mas da obtenção de uma versão final adequada e congruente com a original^(15)^. A tradução exige equivalências semânticas, idiomáticas, conceituais, linguísticas, experienciais e contextuais sobre o construto adaptado^(8)^.

O consenso da tradução e retrotradução realizado neste trabalho, baseou-se na premissa de simplificação sintática, adotando a estratégia de reduzir a complexidade do texto sem modificar seu conteúdo, com o intuito de eliminar redundâncias, ampliar a clareza da mensagem e facilitar o entendimento da informação^(15,16)^, possibilitando sua compreensão por professores de diferentes níveis de ensino.

No processo de tradução inicial, observou-se significativa variação nas versões propostas pelos tradutores, sobretudo nos itens relacionados à intensidade vocal e ao desconforto físico associado ao uso prolongado da voz. Itens como os de número 8 e 11 destacaram-se por divergências quanto à terminologia empregada, refletindo a complexidade inerente à tradução de expressões subjetivas associada à percepção vocal. Essas discrepâncias demandaram intervenções pontuais do comitê de especialistas, que, com base em critérios de clareza, relevância semântica e familiaridade cultural, realizou ajustes específicos para preservar o sentido original dos itens e facilitar sua compreensão por professores brasileiros.

As decisões tomadas pelo comitê refletiram uma atenção especial à adequação linguística da escala. Foram realizadas alterações sintáticas e lexicais, sobretudo nos itens 10, 13, 14 e 16, para simplificar a redação e garantir maior acessibilidade. Foram priorizados ajustes na ordem e na formulação das afirmações de cada item, considerando que a consistência na escrita pode influenciar as respostas dos professores, ao facilitar a compreensão e a escolha das alternativas^(17)^. Ressalta-se que os itens considerados inicialmente como pouco relevantes foram mantidos na versão final, em virtude de abordarem aspectos pertinentes ao construto da fatigabilidade vocal, o que reforça o compromisso metodológico com a integridade conceitual do instrumento.

Os itens 8 e 11 apresentaram divergências de conteúdo entre as traduções realizadas, sendo necessária a reformulação da escrita durante o consenso das traduções. No item 8, observou-se uma duplicidade de significado, uma vez que os termos “voz irritada” e “garganta irritada” remetem a percepções distintas. Optou-se por manter o termo “garganta”, por se referir a uma sensação física, ao passo que “voz irritada” está relacionada a alterações na qualidade vocal. Já no item 11, houve divergência ao comparar os termos “garganta irritada” e “pescoço irritado”, considerando que “garganta” se refere a sensações diretamente ligadas ao trato vocal, enquanto “pescoço” está associado a sintomas decorrentes de esforço físico relacionado à fala. Diante disso, optou-se por manter o termo “pescoço”.

Em relação à aceitabilidade dos itens da escala, a maioria dos professores considerou 13 itens como altamente relevantes: 1, 2, 3, 4, 5, 6, 7, 8, 9, 10, 11, 12 e 15. Esses itens estão majoritariamente associados às sensações percebidas durante o uso da voz. O elevado índice de relevância atribuído à maioria dos itens pelos professores participantes reforça a aceitabilidade e a pertinência do conteúdo da escala.

Os itens 13, 14, 16 e 17, embora tenham apresentado os maiores índices de irrelevância ou necessidade de revisão, foram mantidos na versão final por abordarem aspectos essenciais à detecção da fatigabilidade vocal em professores, além de não terem sido considerados irrelevantes pela maioria dos avaliadores. Esses itens contemplam estratégias de recuperação vocal e limitações decorrentes do uso excessivo da voz, dimensões que, por serem menos perceptíveis de forma imediata, podem ter sido avaliadas como de menor importância pelos participantes.

Apesar disso, sua inclusão se mostra relevante, pois tratam de aspectos fundamentais para a compreensão da fatigabilidade vocal, ao investigar comportamentos adotados diante do esforço vocal prolongado e os meios utilizados para a recuperação vocal. Os itens 16 e 17, de forma particular, são considerados pelos autores do estudo original como fundamentais para quantificar o tempo de recuperação, um aspecto que distingue a fadiga vocal da fatigabilidade vocal (“16- Preciso dormir uma noite inteira para deixar de sentir desconforto ao falar”; “17- Preciso de dois a três dias falando pouco ou ficando em silêncio para recuperar minha voz”). Considerando que a fadiga está relacionada à exposição vocal contínua e ao descanso inadequado, fatores que, com o tempo, favorecem o agravamento de disfunções vocais, a permanência desses itens na escala se justifica por fornecer dados relevantes não apenas para a identificação precoce da fadiga, mas também para o planejamento de estratégias terapêuticas voltadas à saúde vocal dos professores.

O último aspecto a ser discutido refere-se à necessidade de um ajuste terminológico relacionado ao termo utilizado para o público-alvo da escala. A substituição do termo “docente” por “professor” foi fundamentada pelo princípio da equivalência cultural. Embora semanticamente semelhantes, os termos apresentam diferenças em seu uso e reconhecimento no contexto brasileiro, sendo “professor” amplamente utilizado em diferentes níveis de ensino e presente de forma mais frequente na literatura nacional sobre saúde vocal. Tal decisão, validada pelo comitê de especialistas, foi considerada fundamental para a otimização da clareza e da identificação dos participantes com o propósito da escala, sem prejuízo à fidelidade conceitual da versão original.

Com a conclusão da adaptação transcultural para o português brasileiro, a EFV-P será submetida ao processo de validação pelos autores deste estudo, a fim de assegurar as propriedades psicométricas de validade, fidedignidade e precisão da versão adaptada, viabilizando sua aplicação em pesquisas e na prática clínica.

As demais etapas de validação do instrumento deverão ser direcionadas à investigação das propriedades de validade e confiabilidade da versão brasileira da escala, com o intuito de confirmar sua robustez para uso clínico e em contextos de pesquisa. Tal processo permitirá o uso desse instrumento como ferramenta auxiliar na avaliação e intervenção fonoaudiológica de professores, considerando que constituem uma categoria profissional vulnerável a distúrbios vocais, os quais podem comprometer a qualidade de vida, a comunicação e a satisfação no exercício da profissão. A identificação da fatigabilidade vocal por meio da EFV-P possibilitará aos fonoaudiólogos a implementação de medidas preventivas, evitando a progressão para quadros mais graves de disfonia^(5,6)^.

## CONCLUSÃO

A EFV-D foi adaptada transculturalmente para o português brasileiro, originando a versão intitulada "Escala de Fatigabilidade Vocal em Professores (EFV-P)”. As etapas de tradução, retrotradução, análise e avaliação semântica por um comitê de especialistas permitiram assegurar a equivalência conceitual e a clareza dos itens, respeitando as particularidades linguísticas e culturais da população-alvo. Recomenda-se, em estudos futuros, a realização de análises psicométricas para verificar a validade e a confiabilidade da versão final da escala.

## Apêndice 1 Versão transculturalmente adaptada da EFV-D para o português brasileiro, intitulada Escala de Fatigabilidade Vocal em Professores (EFV-P)

Escala De Fatigabilidade Vocal Em Professores (EFV-P)

As frases abaixo apresentam alguns sintomas frequentemente associados a problemas de voz. Assinale a resposta que indica o quanto você apresenta o mesmo sintoma

**Table:**

	Nunca	Quase nunca	Às vezes	Quase sempre	Sempre
**Fator 1 - Fadiga no rendimento vocal**					
1. Depois de mais de duas horas falando em forte intensidade, não consigo continuar em tarefas de grande exigência vocal.	0	1	2	3	4
2. Depois de mais de duas horas falando em forte intensidade, sinto a voz cansada.	0	1	2	3	4
3. Depois de mais de duas horas falando em forte intensidade, percebo uma crescente sensação de esforço vocal.	0	1	2	3	4
4. Depois de mais de 2 horas falando em forte intensidade, minha voz fica rouca.	0	1	2	3	4
5. Depois de mais de 2 horas falando em forte intensidade, tenho que fazer esforço para continuar falando como estava fazendo.	0	1	2	3	4
6. Depois de mais de duas horas falando em forte intensidade, sinto dificuldade para continuar projetando a voz.	0	1	2	3	4
7. Depois de mais de duas horas falando em forte intensidade, sinto minha voz fraca.	0	1	2	3	4
8. Depois de mais de 2 horas falando em forte intensidade, sinto a garganta irritada.	0	1	2	3	4
9. Depois de mais de 2 horas falando em forte intensidade, sinto desconforto no pescoço, na garganta ou na língua.	0	1	2	3	4
10. Depois de mais de duas horas falando em forte intensidade, sinto dificuldade para falar baixo.	0	1	2	3	4
11. Depois de um dia inteiro usando a voz, sinto desconforto ou dor no pescoço.	0	1	2	3	4
**Fator 2 - Condutas reparadoras**					
12. Depois de um tempo utilizando a voz, costumo limitar a quantidade de fala.	0	1	2	3	4
13. Evito situações sociais em que sei que vou falar muito.	0	1	2	3	4
14. Sinto que não consigo falar com minha família depois de um dia de trabalho.	0	1	2	3	4
15. Preciso ficar em silêncio e me hidratar por mais de uma hora para deixar de sentir esforço para falar.	0	1	2	3	4
16. Preciso dormir uma noite inteira para deixar de sentir desconforto ao falar.	0	1	2	3	4
17. Preciso de dois a três dias falando pouco ou ficando em silêncio para recuperar minha voz.	0	1	2	3	4
