# Supplementary material for: ERRATUM: Cross-cultural adaptation of the Escala De Fatigabilidad Vocal en Docentes (EFV-D) to brazilian portuguese
Source: Codas. 2026 Sep 25;38(5):e20260189. doi: 10.1590/2317-1782/e20260189en (PMC13644368; doi:10.1590/2317-1782/e20260189en)
Supplement: Translation

# Translation: ERRATA: Adaptação transcultural da *Escala de Fatigabilidad Vocal en Docentes (EFV-D)* para o português brasileiro

**DOI:** 10.1590/2317-1782/e20260189pt

Devido a erro honesto dos autores, o artigo “Adaptação transcultural da Escala de *Fatigabilidad Vocal en Docentes* (*EFV-D*) para o português brasileiro” (DOI https://doi.org/10.1590/2317-1782/e20250426pt), presente em CoDAS 2026;38(5):e20250426, foi publicado sem a identificação de todos os autores e com ordem de autoria incorreta.

**Onde antes se identificava:**

Maria Eduarda Oliveira de Figueiredo, Priscila Oliveira, Thays Vaiano, Thiago Henrique de Pontes Ferreira e Larissa Nadjara Alves Almeida.

**Agora, identifica-se:**

Maria Eduarda Oliveira de Figueiredo, Larissa Nadjara Alves Almeida, Thays Vaiano, Thiago Henrique de Pontes Ferreira, Silvia Contreras-Regatero e Priscila Oliveira.

Os autores pedem desculpas pelo erro.

**Silvia Contreras-Regatero**

**ORCID:** https://orcid.org/0000-0002-1589-4903

**Afiliação:** Department of psychology and speech language pathology, Universitat Ramon Llull, Barcelona, Espanha.

**Contribuição:** Validação e Redação – revisão e edição.

Editor:

Aline Mansueto Mourão
